# Preliminary report of surface electrogastrography in critically ill septic patients after resuscitation

**DOI:** 10.1186/cc10767

**Published:** 2012-03-20

**Authors:** C Mancilla, R Galvez, G Landskron, E Tobar, A Madrid

**Affiliations:** 1 Hospital Clinico Universidad de Chile, Santiago, Chile

## Introduction

Impaired gastrointestinal motility is common in critically ill patients. Multiple conditions such as shock with diminished splanchnic perfusion, surgery, fluid overload, intra-abdominal hypertension, and drugs are responsible for this phenomena. Assessing gastric motility in this setting is complex. Surface electrogastrography (sEGG) is a recent noninvasive technique that determines basal and postprandial gastric motility [1]. Our aim is to study basal gastric motility in critically ill septic patients in the post-resuscitative phase, by sEGG.

## Methods

Eligible patients were those admitted to the ICU with diagnosis of septic shock as stated by the Sepsis Conference 2001 [2]. At the moment of the study the patients were in the post-resuscitative phase, defined as normal clinical and laboratory perfusion parameters. sEGG is a noninvasive technique that uses skin abdominal electrodes to record myoelectrical stomach activity. The basal slow wave originates in the proximal stomach and propagates to the antrum with a frequency of approximately 3 cycles per minute (cpm). Basal activity below 2.4 cpm is defined as bradygastria and above 3.7 cpm as tachygastria [1]. Data were correlated with severity scores, lactate levels, and doses of sedatives. The study was approved by the Ethics Committee of the Hospital Clínico Universidad de Chile.

## Results

We recruited 16 patients (10 females). Mean age 62 years (50 to 76) (*P *= 0.8). APACHE II score 25 (19 to 28) (*P *= 0.4) and SOFA score 9 (7 to 11) (*P *= 0.29). Lactate at admission 3.8 mmol/l (1.2 to 6.5) (*P *= 0.72). Fentanyl total dose 172.7 μg/kg (59 to 256.6) (*P *= 0.91) and midazolan total dose 3.4 mg/kg (0.1 to 3.1) (*P *= 0.07). We obtained a reliable register in all the patients and found six patients with bradigastria, three with tachygastria and nine with normal motility. In this small sample size study there was a trend to bradygastria in relation to high total doses of midazolam.

## Conclusion

sEGG is a feasible technique in critically ill septic patients. In the post-resuscitative phase 43.8% of patients present normal gastric motility, and 37% showed bradygastria. Future research is warranted in order to find risk factors of gastrointestinal dismotility.
